# Oxide Semiconductor Thin‐Film Transistors for Low‐Power Electronics

**DOI:** 10.1002/advs.202522487

**Published:** 2026-01-21

**Authors:** Shuhui Ren, Qi Huang, Dingwei Li, Saisai Wang, Yitong Chen, Huihui Ren, Yan Wang, Yingjie Tang, Bowen Zhu

**Affiliations:** ^1^ Zhejiang Key Laboratory of 3D Micro/Nano Fabrication and Characterization Department of Electronic and Information Engineering School of Engineering Westlake University Hangzhou Zhejiang China; ^2^ Westlake institute For Optoelectronics Hangzhou China

**Keywords:** low‐power electronics, oxide semiconductor, thin‐film transistor

## Abstract

Low power consumption has become an essential criterion in the development of next‐generation electronics, driven by the growing adoption of Internet of Things, wearables, and portable platforms. Oxide semiconductor thin‐film transistors (TFTs) have become most promising candidates for next‐generation low‐power electronics due to their wide band‐gap, low leakage current, high mobility, steep subthreshold swing, and compatibility with low‐temperature flexible processing. In this review, recent advances in the use of oxide TFTs for low‐power electronics are systematically summarized. First, the inherent advantages of oxide semiconductor materials over other commonly used materials (e.g., amorphous hydrogenated silicon, low temperature polycrystalline silicon, organic semiconductors, etc.) for realizing low power consumption are demonstrated. Then, strategies to reduce power consumption are further discussed, including interface engineering, such as the novel source‐gated transistors, and structural engineering, such as dual‐gate and underlap designs. Finally, a comprehensive review of oxide TFTs for various low‐power electronics applications, including logic circuits, active‐matrix arrays, flexible electronics, monolithic 3D integration, and neuromorphic computing, is presented, demonstrating their great potential in future low‐power and flexible electronic systems.

## Graphical abstract

This review explores the progress of oxide semiconductor thin‐film transistors in low‐power electronics. It illustrates the inherent material advantages of oxide semiconductor, which enable it to meet the low‐power requirements. It also discusses current strategies for reducing power consumption, including interface and structure engineering. Finally, it illustrates their applications in logic circuits, active‐matrix arrays, flexible electronics, 3D integration, and computing.

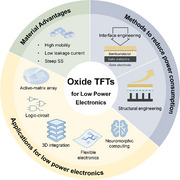

## 1 Introduction

Low‐power electronics underpin the rapid development of the Internet of Things (IoT), wearable devices, and portable computing [1, 2]. The central objective is to sustain long‐term, reliable operation under energy‐constrained conditions, which requires extremely low static and dynamic power consumption [3, 4, 5]. Beyond energy efficiency, these emerging applications often demand electronics that are low‐cost and scalable, with compatibility for large‐area fabrication and flexible substrates. However, conventional silicon‐based complementary metal‐oxide‐semiconductor (CMOS) technologies currently rely on complex and high‐cost fabrication processes involving high‐temperature treatments and rigid wafers, which are not well‐suited for these application scenarios [6, 7]. This difference in performance requirements motivates increasing interest in alternative transistor technologies that prioritize energy efficiency, mechanical flexibility and process scalability.

Oxide semiconductor thin‐film transistors (TFTs) have emerged as a promising solution to meet these evolving demands [8, 9, 10]. Unlike conventional silicon‐based technologies, oxide TFTs utilize amorphous or polycrystalline metal oxide semiconductors, such as indium gallium zinc oxide (IGZO), as the active channel material [11]. These materials offer an attractive combination of properties, including medium to high carrier mobility, low intrinsic leakage current (due to their wide band‐gap), and steep subthreshold swing (SS), which can effectively reduce power consumption [12, 13]. Meanwhile low‐temperature process compatibility enables integration on flexible polymer substrates, breaking through rigid silicon‐based limitations [14, 15]. In addition, such materials can achieve excellent large‐area uniformity and fine patterning capabilities supported by advanced deposition processes, laying the foundation for large‐scale flat‐panel display and integrate circuit applications [16, 17, 18].

**FIGURE 1:**
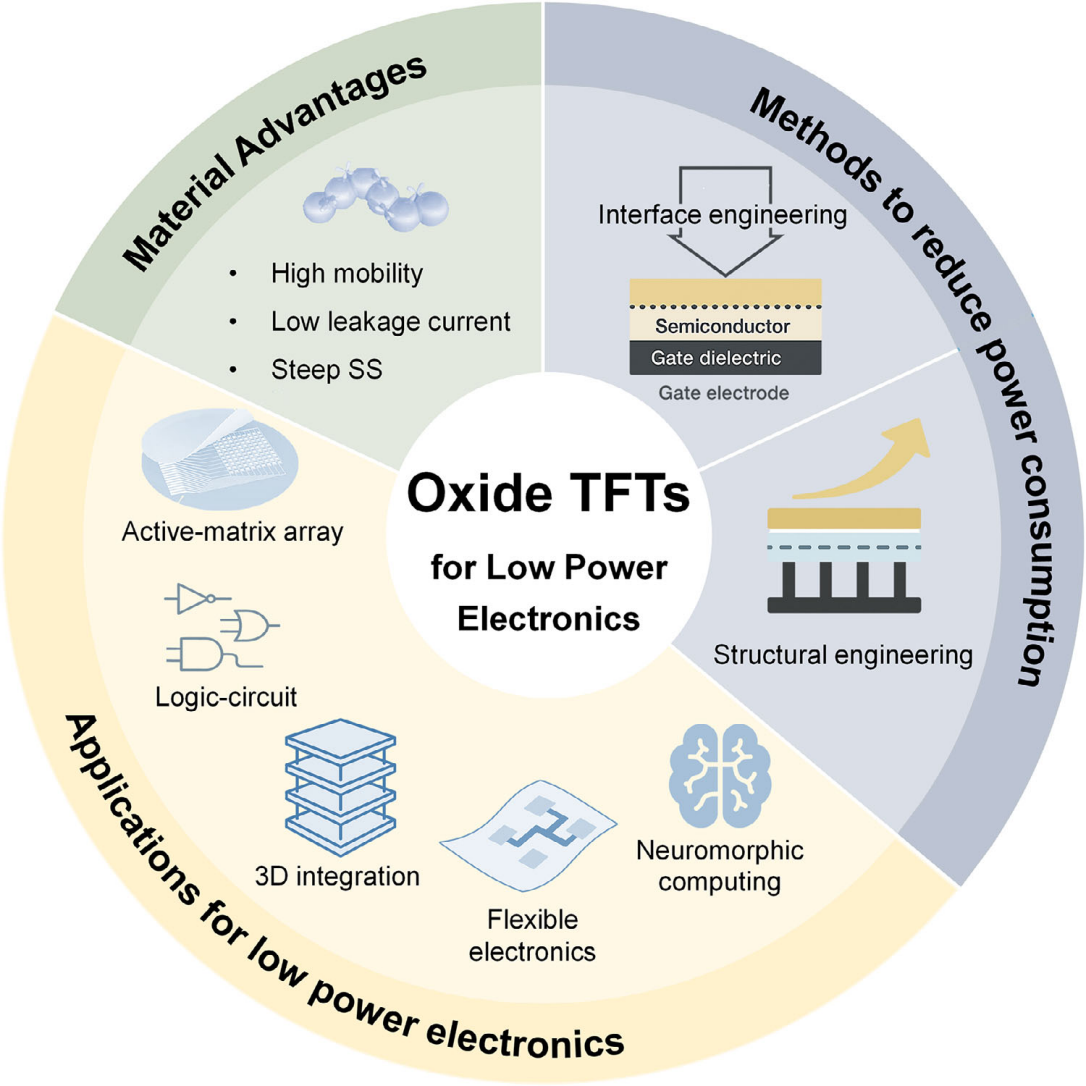
Schematic diagram illustrating oxide semiconductor TFTs for low‐power electronics.

In this review, we systematically summarize the recent research progress of oxide semiconductor TFTs in the field of low‐power electronics, as shown in Figure 1. First, the inherent advantages of oxide TFTs in low‐power electronics are elucidated in terms of material properties and key performance metrics (such as mobility, SS, leakage current, etc.). Subsequently, the current research progress on strategies to reduce power consumption in oxide TFTs, including interface optimization and structural engineering, is further discussed. Finally, the representative applications of oxide TFTs in logic circuits, active‐matrix arrays, flexible electronics, 3D integrated circuits, and neuromorphic computing systems are presented, which demonstrate the technological potentials and future directions of oxide TFTs in the field of low‐power electronics.

## 2 Advantages of Oxide Semiconductor TFT for Low‐Power Electronics

Low power consumption is one of the most critical requirements for TFTs in circuit applications, especially in energy‐constrained systems such as wearable devices, IoT nodes, and edge computing platforms. The total power consumption (P) consists of both dynamic and static components [19]. The dynamic power P_dynamic_ is primarily determined by:

(1) $$P_{\mathrm{dynamic}}=C_{total}\cdot V_{dd}^{2}\cdot f$$

where C_total_ is the total load capacitance, V_dd_ is the supply voltage, and f is the operation frequency. This power reflects the energy required to charge and discharge internal and external capacitive nodes during switching events [20, 21]. Since the dynamic power consumption increases with the square of V_dd_, lowering V_dd_ is an effective strategy to reduce the power consumption.

In parallel, the static power P_static_ is dictated by the off‐state leakage current I_off_, which can be calculated by:

(2) $$P_{\mathrm{static}}=I_{off}\cdot V_{dd}$$

Here, off‐state leakage current I_off_ represents the primary source of energy loss in standby systems, and minimizing I_off_ is essential to improve the overall system efficiency [22, 23]. To fully exploit the potential of TFTs in ultra‐low‐power electronics, both dynamic and static power must be simultaneously optimized.

**FIGURE 2:**
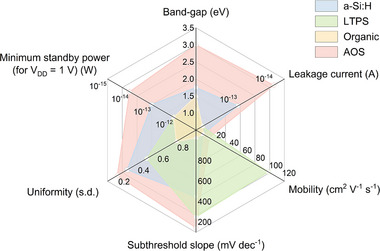
The performance comparison of the main channel materials of TFTs, including a‐Si:H, LTPS, organic, and AOS.

Compared to traditional TFT materials (e.g., amorphous hydrogenated silicon (a‐Si:H), low temperature polycrystalline silicon (LTPS), organic semiconductors), amorphous oxide‐semiconductor (AOS) TFTs, as an emerging class of semiconductor technologies, exhibit unique competitive advantages for low‐power electronic applications [24, 25]. The key performance comparison of TFT devices based on several major active layer materials is shown in Figure 2 and Table 1, and it can be clearly found that oxide semiconductor TFTs offer the following advantages:

**TABLE 1:** Comparison of performances of the main TFT technologies.

	a‐Si:H	LTPS	Organic	AOS
Band‐gap (eV)	1.7‐1.8	∼1.1	1‐2	> 3
Leakage current (A)	10^−13^	10^−12^	10^−13^‐10^−11^	≪10^−14^
Mobility (cm^2^ V^−1^ s^−1^)	0.5	∼100	∼1	∼10
SS (mV dec^−1^)	400–500	200‐300	50‐2000	90‐200
TFT uniformity σ (s.d.)	Good (σ < 0.3)	Medium (σ < 0.5)	—	Very good (σ < 0.2)
Minimum standby power (for V_dd_ = 1 V) (W)	∼10^−13^	∼10^−12^	∼10^−12^	<10^−14^
Cost efficiency	Low	High	Low	Low
Process temperature (°C)	250‐350	350‐550	< 130	≤ 350
Process complexity	Low	High	Low	Low
Large‐area uniformity	Good	Poor	Poor	Good

1) Wide band‐gap and ultra‐low leakage current

AOS materials generally have a wide band‐gap (> 3 eV), which is significantly greater than that of a‐Si:H (1.7‐1.8 eV), LTPS (∼1.1 eV), and organic semiconductors (1‐2 eV) [26, 27]. This wide band‐gap is associated with low intrinsic carrier concentrations and enhanced environmental stability. As a result, oxide TFTs exhibit extremely low off‐state leakage currents, often below 10^−14^ A, which is at least an order of magnitude lower than other TFT technologies [28]. This suppression of leakage current is critical for reducing standby power consumption, especially in battery‐powered or always‐on applications. For a supply voltage V_dd_ of 1 V, the static power consumption of oxide TFTs is less than 10^−14^ W, which is superior to that of a‐Si:H, LTPS, and organic TFTs [29].

2) Steep SS and high mobility for efficient switching

The SS reflects how efficiently a transistor transitions from the off‐state to the on‐state in response to gate voltage variation. Oxide TFTs typically exhibit SS values in the range of 90–200 mV/dec, markedly better than a‐Si:H (400‐500 mV/dec) and most organic semiconductors (often exceeding 500 mV/dec) [30]. In terms of the mobility, the typical mobility of AOS‐based TFTs is up to 10–50 cm^2^ V^−1^ s^−1^, which is higher compared to conventional a‐Si:H TFTs (with mobility usually below 1 cm^2^ V^−1^ s^−1^) and organic TFTs (around 1 cm^2^ V^−1^ s^−1^) [30, 31, 32]. The advantage of steep SS and high mobility enables oxide TFTs to operate effectively at low V_dd_ while maintaining fast switching speeds, thereby simultaneously reducing V_dd_ requirements and dynamic power consumption [33, 34].

3) High process compatibility and excellent TFT uniformity

In contrast to LTPS, which requires laser annealing or high‐temperature crystallization, oxide semiconductor TFTs can typically be prepared at lower process temperatures (≤ 350°C), and they are more compatible with flexible substrates and back‐end‐of‐line (BEOL) integration [35, 36, 37]. In addition, due to its amorphous nature and low temperature deposition process, oxide TFTs achieve excellent device uniformity (σ < 0.2) over large areas. This superior uniformity is critical for large‐area sensor arrays and high‐resolution display backplanes. Simultaneously, oxide TFTs have low‐cost efficiency and reduced process complexity [24, 38, 39].

Compared with other emerging technology platforms such as 2D material TFTs and carbon nanotube TFTs, AOS TFTs offer superior control over static power consumption due to their extremely low off‐state current. Furthermore, they demonstrate greater industrialization potential than 2D materials and carbon nanotube technologies in terms of process maturity, large‐area uniformity, and manufacturing costs [40, 41, 42].

## 3 Methods for Achieving Low Power Consumption

Although oxide TFTs inherently exhibit greater potential for low power consumption compared to other TFT technologies, researchers nevertheless persistently devise strategies to further reduce power dissipation in oxide‐based devices and circuits. Recent advances predominantly fall into two categories: interface engineering and structural engineering. This section focuses on progress in these two key directions.

### 3.1 Optimization of Interface Engineering

**FIGURE 3:**
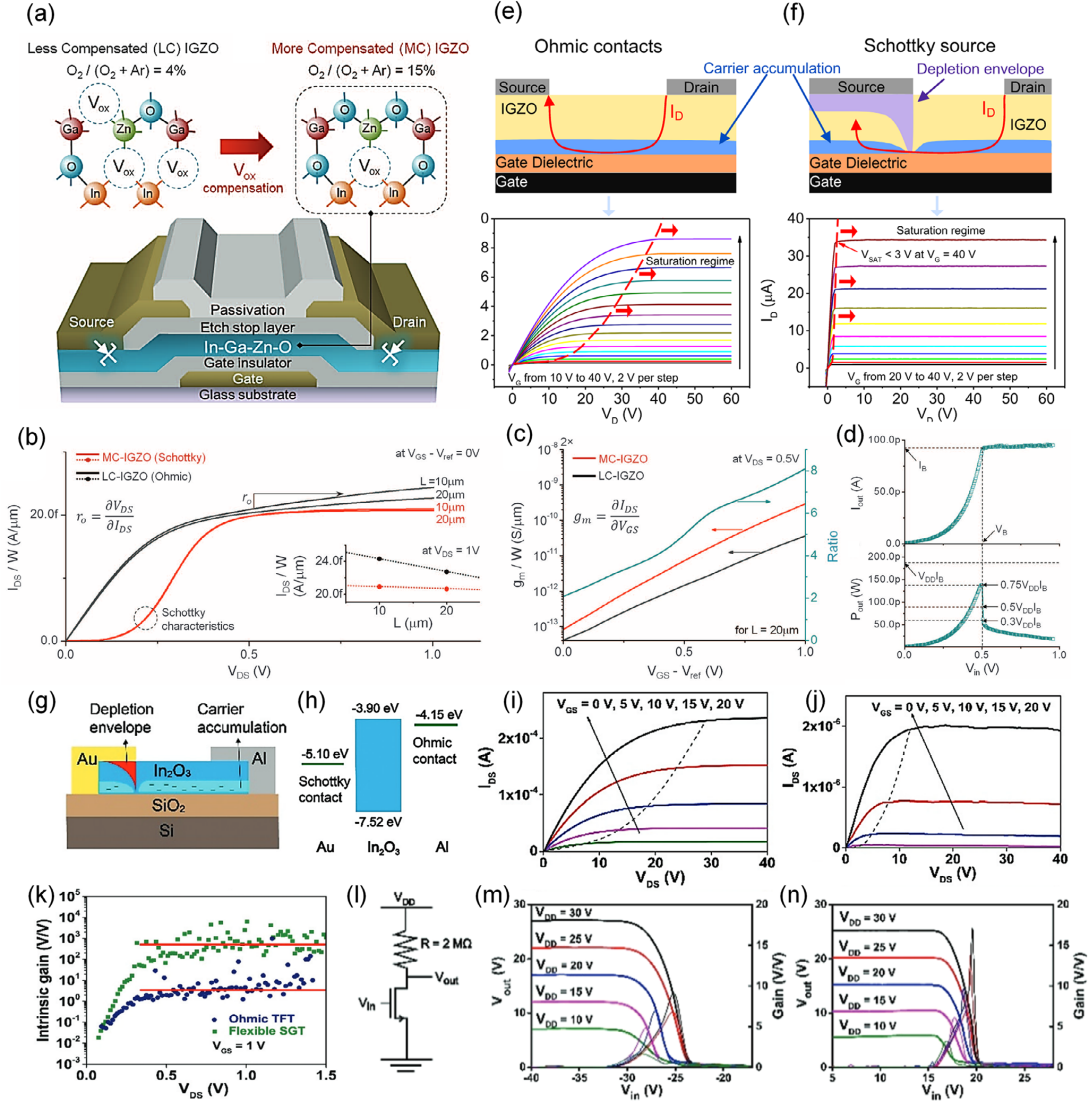
(a) Schematic illustration of IGZO TFTs fabricated under different oxygen partial pressures during sputtering, leading to less compensated (LC) and more compensated (MC) IGZO films. (b) Output characteristics of LC‐IGZO and MC‐IGZO TFTs showing Schottky versus ohmic behavior at various channel lengths. (c) Extracted output resistance and transconductance values of MC‐IGZO and LC‐IGZO TFTs. (d) Voltage gains and output power consumption characteristics of the Schottky barrier TFT‐based inverter circuit. Reproduced with permission [29]. Copyright 2016, The American Association for the Advancement of Science. (e, f) Device schematics and output characteristics comparing conventional ohmic‐contact TFTs (e) and SGTs (f), highlighting differences in current saturation behavior. Reproduced with permission [49]. Copyright 2019, PNAS. (g) Device structure of a flexible In_2_O_3_ SGT with asymmetric Au/Al source/drain contacts. (h) Energy band diagrams of In_2_O_3_ with Au and Al contacts, showing Schottky and ohmic contact formation. (i, j) Output characteristics of In_2_O_3_ TFTs with symmetric (Al/Al) and asymmetric (Au/Al) contacts, respectively. (k) Intrinsic gain comparison between ohmic TFTs and flexible SGTs. (l) Schematic of the inverter circuit based on SGT devices. (m, n) Voltage transfer characteristics and corresponding voltage gain curves for ohmic TFT‐ and SGT‐based inverters at different V_dd_. Reproduced with permission [53]. Copyright 2019, PNAS.

Interface engineering plays a crucial role in improving the performance of oxide TFTs and enabling low‐power operation, which is a key strategy to improve device performance by precisely tuning material interfaces such as electrode‐semiconductor contacts [43, 44]. One prominent approach involves introducing a Schottky barrier at the source contact, which effectively suppresses the saturation current to reduce power dissipation [45]. As illustrated in Figure 3a, Lee et al. developed a Schottky barrier IGZO TFT with ultra‐low power consumption by modulating the oxygen partial pressure (P_ox_) during the sputtering deposition of the IGZO channel layer [29]. The device exhibits excellent performance in the deep subthreshold regime (near off‐state), achieving stable operation at supply voltages below 1 V and power consumption controlled within 1 nW, which provides a new strategy for realizing low‐power oxide TFTs. [46, 47, 48] In the measured output characteristics (Figure 3b), the Schottky barrier TFT shows a much flatter output curve and higher output resistance compared to the TFT with conventional ohmic contacts. In addition, the Schottky barrier TFTs demonstrate higher transconductance than the ohmic contact device (Figure 3c), enabling low‐voltage operation and high intrinsic gain. Consequently, the voltage gain of the inverter circuit based on this Schottky barrier TFT exceeds 220, and the output power consumption (P_out_) is less than 150 pW (Figure 3d) due to the extremely low operating current, thereby validating a practical pathway to realizing nanowatt‐scale logic circuits.

Zhang et al. also designed TFTs with extremely high intrinsic gain by leveraging a conduction mechanism recently discovered by their group in reverse‐biased thin‐film Schottky diodes [49]. For conventional TFT and proper operation, the source/drain contacts must be ohmic (i.e., low resistance) as shown in Figure 3e. In contrast, in source‐gated transistor (SGT) (Figure 3f) the source contacts are replaced with Schottky barriers, regulating the current at the source stage [50, 51, 52]. The authors modulated the oxidation of Pt contact electrodes by introducing oxygen content and controlling sputtering power during the deposition process, thereby regulating the Schottky barrier at the source terminal. The output curves demonstrate that SGT achieves current saturation at significantly lower drain voltages, and its saturation current can be reduced by two orders of magnitude compared to conventional TFT. As a result, the more excellent saturation characteristics lead to an intrinsic gain as high as 29,000, and the smaller saturation voltage and lower saturation current achieved by the SGT can result in a significant reduction in power consumption.

Li et al. fabricated a flexible Schottky barrier SGT based on solution‐processed indium oxide (In_2_O_3_) semiconductors [53]. As shown in Figure 3g, the In_2_O_3_ SGT utilized asymmetric source/drain electrodes, specifically designed to form a Schottky barrier at the source and an ohmic contact at the drain. This design relies on specific energy band alignment at the metal‐semiconductor interfaces: the high work function of Au (5.10 eV) combined with the electron affinity of In_2_O_3_ (3.90 eV) creates a Schottky barrier of approximately 1.20 eV at the source contact, whereas the lower work function of Al (4.15 eV) facilitates the formation of an ohmic contact at the drain, as illustrated in the energy band diagrams of Figure 3h. The output curves (Figure 3i,j) reveal that the SGT achieves current saturation at a substantially lower drain voltage compared to a symmetric ohmic‐contact device. This fast saturation behavior contributes to low power dissipation. The power consumption (P_sat_ = I_DS_ × V_DS_/(W × L)) of flexible SGT is two orders of magnitude lower than that of the ohmic counterpart, demonstrating the advantages of SGTs in electronic devices with low‐power requirements. Further analysis reveals that the SGT achieves a much higher intrinsic gain, as shown in Figure 3k, which indicates an intrinsic gain of 792 for the SGT, almost 40 times higher than that of the ohmic device (gain ≈ 20). When integrated into a basic inverter circuit (Figure 3l), the SGT‐based inverters achieve higher voltage gain than ohmic TFT‐based inverters at the same supply voltage (Figure 3m,n). These excellent properties highlight the circuit application potential of SGTs.

In summary, interface engineering plays a pivotal role in achieving ultra‐low power consumption in oxide TFTs by effectively modulating the semiconductor‐electrode interfaces. Strategies such as partial oxygen pressure modulation, Schottky barrier engineering, and asymmetric contact design have demonstrated significant improvements in key device metrics, including reduced saturation voltage and current, enhanced intrinsic gain, and minimized leakage current [45, 54, 55, 56]. These advancements establish a solid foundation for further optimizing device architectures and circuit designs to meet the stringent power requirements of next‐generation flexible and wearable electronics.

### 3.2 Optimization of Structure Engineering

Structure engineering is a key approach to improving the electrical performance and reliability of devices by designing and optimizing the device geometry [57, 58]. Device characteristics such as carrier mobility, leakage current, switching behavior, and operating stability can be significantly improved by precisely tuning the geometrical configuration, microstructure and interface arrangement, which provides an effective way to optimize the electrical characteristics of oxide TFTs [59, 60].

**FIGURE 4:**
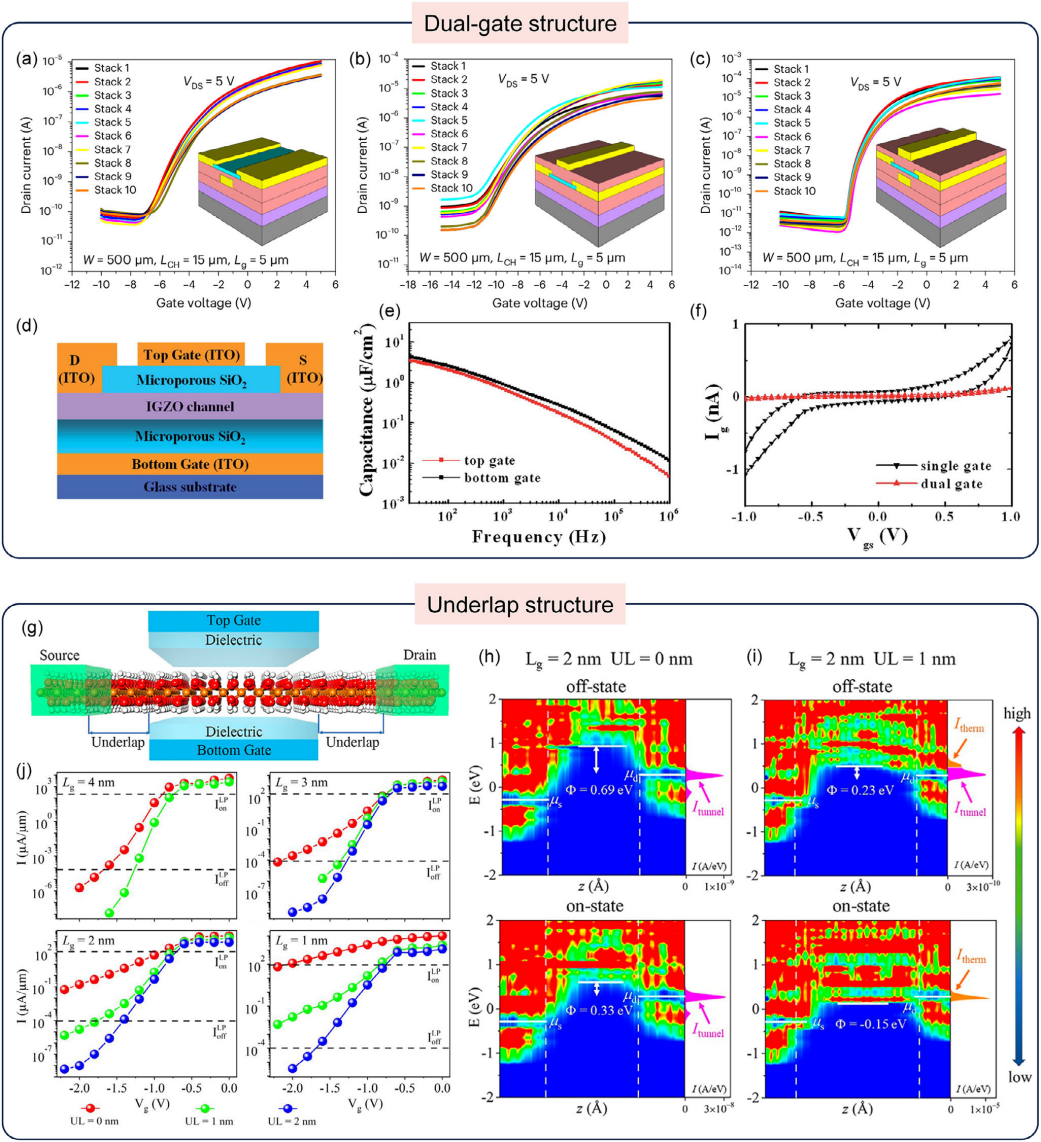
(a–c) Transfer characteristics of the 10‐stack BG devices (a); TG devices (b) and DG devices (c). Reproduced with permission [61]. Copyright 2024, Springer Nature. (d) Schematic structure of the DG IGZO TFT with the driving bottom gate and the control top‐gate. (e) Specific gate capacitance of the TG and BG dielectrics of the DG TFTs. (f) Leakage current comparison between the single gate and DG TFTs. Reproduced with permission [62]. Copyright 2020, The Royal Society of Chemistry. (g) Schematic diagram of the ultrathin In_2_O_3_ MOSFET device. (h, i) Local density of states and spectral current distribution of the ultrathin In_2_O_3_ MOSFETs at gate lengths L_g_ = 2 nm with UL = 0 nm (h) and UL = 1 nm (i). The electron barrier height Φ is shown, µs and µ_d_ denote the Fermi levels of the source and drain, respectively. The white dashed line indicates the boundary between the electrode and channel regions. (j) Transfer characteristics of the n‐type ultrathin In_2_O_3_ field effect transistors for low‐power applications at Lg = 4 nm, 3 nm, 2 nm and 1 nm with varying underlap lengths (0‐2 nm). The black dashed lines indicate the low‐power requirements for on‐state and off‐state currents from the International Technology Roadmap for Semiconductors. Reproduced with permission [63]. Copyright 2024, American Chemical Society.

Another significant direction is the development of dual‐gate (DG) architectures to strengthen gate control. Yuvaraja et al. proposed a method to fabricate the vertically integrated 10‐stack dual‐gate (DG) In_2_O_3_ transistors [61]. As evidenced by the transfer characteristics (Figure 4a–c), the DG configuration significantly outperforms single‐gate devices, reducing the off‐state current by two orders of magnitude, thereby lowering static power consumption. Simultaneously, DG TFT improves mobility and reduces SS, which enables the DG TFT to operate at a lower V_dd_, and further reduces the dynamic power consumption. In addition, Dou et al. fabricated a DG amorphous IGZO (a‐IGZO) bilayer electric double‐layer (EDL) TFT, as shown in Figure 4d [62]. The top‐gate (TG) and bottom‐gate (BG) dielectrics exhibited high specific capacitance values, enabling a low operating voltage of 1 V as shown in Figure 4e. Figure 4f shows that the leakage current of the DG TFT is less than 0.2 nA, much smaller than that of standard TFTs (1 nA), highlighting their potential for low‐power applications.

Beyond multi‐gate designs, the introduction of an underlap (UL) structure between the gate and source/drain electrodes presents another effective strategy to minimize leakage and enhance device performance [63]. Xu et al. explored this in sub‐5 nm ultrathin In_2_O_3_ metal‐oxide‐semiconductor field‐effect transistors (MOSFETs), as shown in Figure 4g, with UL lengths of 0/1/2 nm between the gate and electrodes. Simulation results (Figure 4h,i) reveal that the UL structure can modulate the tunneling barrier for electrons. In the off‐state, a longer UL increases barrier width, suppressing tunneling current and reducing static power consumption. Concurrently, this design allows for efficient barrier lowering during switching, enabling a high on‐state current. This superior electrostatic control translates directly into improved switching characteristics across scaled gate lengths, as evidenced by the transfer curves in Figure 4j, demonstrating the UL structure's potential for enabling low‐power oxide transistors.

In summary, structure engineering plays a pivotal role in optimizing the electrical performance and power efficiency of oxide TFTs. Strategies such as DG and UL structures have been demonstrated to significantly improve the key device characteristics including carrier mobility, subthreshold swing, and leakage current suppression through precise tuning of geometrical configurations, microstructures, and interfacial arrangements [64, 65, 66]. These innovations not only improve device stability, but also provide effective ways to reduce power consumption, laying a solid foundation for further advancing the development of low‐power, high‐performance electronics such as large‐area displays, wearable devices and high‐speed TFT logic circuits.

## 4 Applications of Oxide TFTs in Low‐Power Electronics

### 4.1 Logic Circuits

Logic circuits play a fundamental role in digital electronics, enabling a wide range of applications from simple computational tasks to complex processing systems [67, 68]. In recent years, the development of TFTs has opened up new possibilities for creating low‐power, high‐performance logic circuits. This section focuses on n‐type metal oxide semiconductor (NMOS) inverters, CMOS inverters and other basic logic gates realized with oxide TFT technologies [69, 70].

**FIGURE 5:**
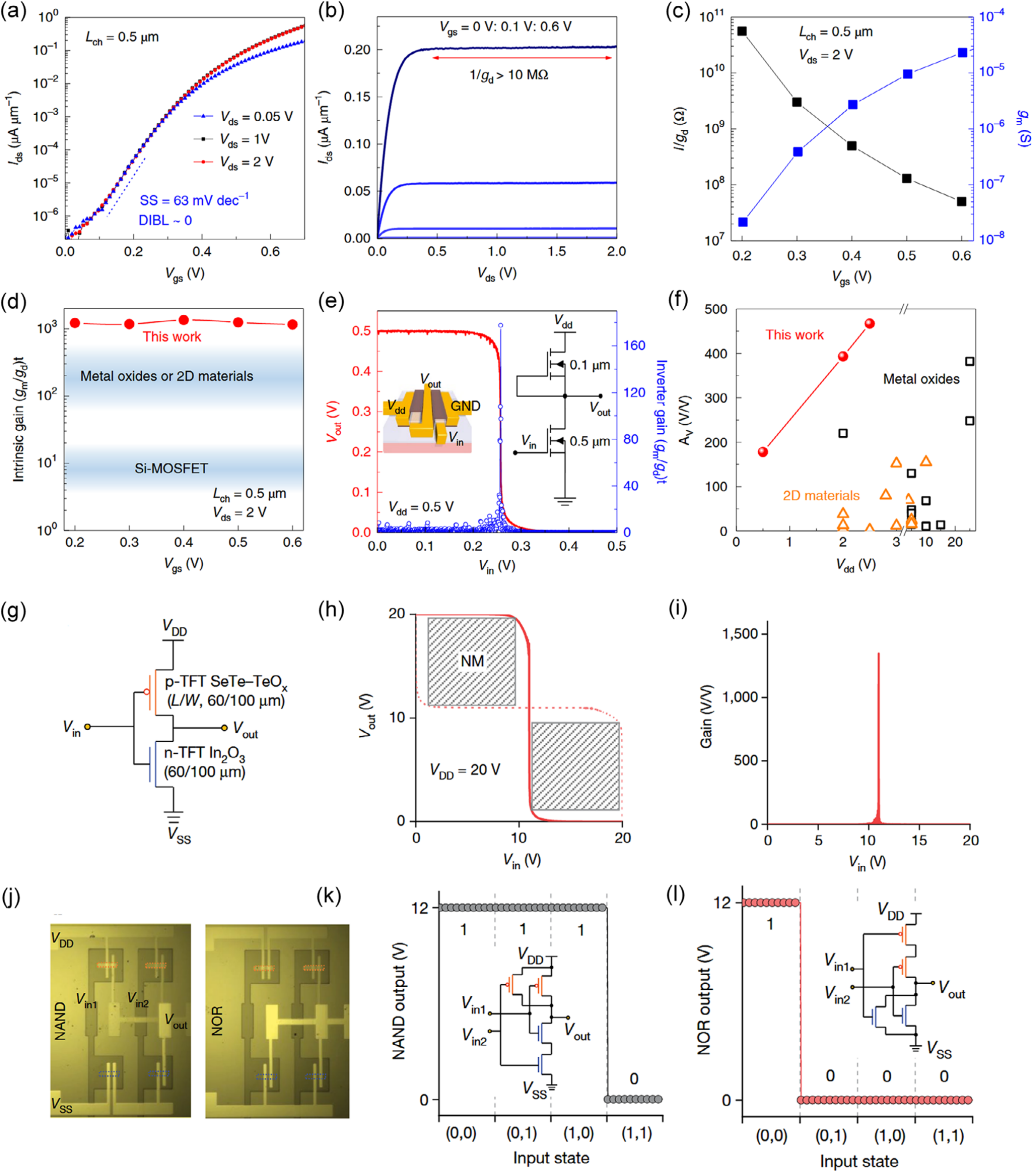
(a,b) Transfer characteristics (a) and Output characteristics (b) of ITO transistors. (c) Extracted values of 1/g_d_ (black squares) and g_m_ (blue squares). (d) Measured intrinsic gain, g_m_/g_d_. (e) Output voltage and inverter gain for the logic inverter. Inset: schematic view of the enhancement‐depletion mode inverter. (f) Benchmark of inverter gain as a function of V_dd_ for inverters based on metal oxides, 2D materials and ITO transistors in this work. Reproduced with permission [71]. Copyright 2019, Springer Nature. (g–i) Performance of integrated CMOS circuits: diagram (g), voltage transfer characteristics and noise margin extraction (h), and gain voltage curves (i) for a complementary inverter based on n‐channel In_2_O_3_ and p‐channel Se‐alloyed Te‐TeO_x_ TFTs at V_dd_ = 20 V. (j–l) Photograph (j), input and output waveforms for complementary NAND (k) and NOR (l) logic gates at V_dd_ = 12 V. The red and blue boxes in (j) indicate the positions of the p‐channel Se‐alloyed Te‐TeO_x_ and n‐channel In_2_O_3_ TFTs, respectively. Reproduced with permission [72]. Copyright 2024, Springer Nature.

]Li et al. demonstrated ultra‐thin ITO transistors with the channel thickness of 4 nm and equivalent oxide thickness of 0.8 nm, where the transfer curve of the device (channel length is 0.5 µm) shows a near‐thermal‐limit SS of 63 mV/dec, and an almost negligible drain‐induced barrier lowering (Figure 5a) [71]. This characteristic results in a high output resistance (above 10 MΩ, Figure 5b), and a large intrinsic gain, defined as A_i_ = g_m_/g_d_, where g_m_ is the transconductance and g_d_ is the output conductance, approaching 1,000 biased at 0.2–0.6 V, as shown in Figure 5c. Notably, the intrinsic gain of this ITO transistor is much higher than that of other metal oxide or 2D material transistors and almost two orders of magnitude higher than that of Si metal‐oxide‐semiconductor FETs (Figure 5d). These device‐level characteristics are particularly relevant to low‐power circuits because they simultaneously suppress standby power loss and help scale down the V_dd_ required for logic operations. Based on this, an enhancement‐depletion mode NMOS inverter achieves full‐swing transfer characteristics at V_dd_ of 0.5 V with a high transfer gain of 178, as shown in Figure 5e, substantially outperforming previously reported metal‐oxide and 2D‐material inverters (Figure 5f). Such high‐gain, low‐V_dd_ operation directly supports aggressive supply‐voltage scaling and thus reduces dynamic power consumption, highlighting the potential of oxide transistors for energy‐efficient logic.

Beyond NMOS architectures, CMOS TFT logic circuits provide another low‐power approach by minimizing static power consumption. Therefore, developing high‐performance p‐channel oxide TFTs is crucial. Liu et al. developed high‐performance and stable p‐channel TFTs using amorphous mixed‐phase semiconductors based on Se‐alloyed Te‐TeO_x_ [72]. Theoretical analysis reveals the existence of delocalized valence band and shallow acceptor states in the tellurium, enabling hole doping and transport. Selenium alloying promotes the connectivity of p‐orbitals, thus achieving high‐performance p‐type TFTs. The average hole mobility is approximately 15 cm^2^ V^−1^ s^−1^, and the on/off current ratio exceeds 10^6^. By integrating the p‐channel Te‐TeO_x_ with n‐channel In_2_O_3_, a CMOS inverter was constructed, as shown in Figure 5g. This CMOS inverter exhibits full‐swing characteristics for fast voltage conversion, as shown in Figure 5h,i. The voltage gain reaches an impressive 1,300 at a V_dd_ of 20 V. The inverter also shows a high noise margin (82% of V_dd_/2), indicating its excellent tolerance to noise and input signal variations in cascaded integrated circuit applications. Additionally, the authors constructed two important logic gates, NAND and NOR, as shown in Figure 5j. Based on the test results shown in Figure 5k,l, these two logic gates function correctly, providing ideal rail‐to‐rail output voltages that correspond to their input states. Furthermore, Table 2 presents a comprehensive comparison of performance and power consumption of the oxide‐based TFT inverters reported in recent studies, demonstrating their compliance with fundamental specifications for low‐power electronic applications [2, 73, 74, 75, 76, 77, 78, 79, 80, 81, 82, 83, 84, 85, 86, 87].

**TABLE 2:** Summary of performance and power consumption of oxide‐TFTs‐based inverters.

Inverter type	Channel material	Gain	V_dd_ (V)	P_static_	P_maximum_	Ref
All‐oxide CMOS	n‐type: ZnO/p‐type: SnO	80	10	0.28 µW	11 µW	[73]
	n‐type: a‐IGZO/p‐type: SnO	226	3	15.6 nW	241.2 nW	[74]
	n‐type: a‐IGZO/p‐type: SnO	4.9	17	32 pW	/	[75]
	n‐type: a‐IGZO/p‐type: SnO	4.2	17	32 pW	/	[2]
	n‐type: In_2_O_3_/p‐type: Cu_2_O	18	1.5	1 nW	/	[76]
	n‐type: IGZO/p‐type: SnO_x_	57	0.5	34 pW	/	[77]
	n‐type: Ga_2_O_3_/p‐type: SnO	149	50	20 nW	84 nW	[78]
	n‐type: ITO/ p‐type: Te‐TeO_x_	132	2	50 pW	134.7 nW	[79]
Hybrid CMOS	n‐type: In_2_O_3_/p‐type: CNT	7.8	0.4	0.6 µW	/	[80]
	n‐type: a‐IGZO/p‐type: CNT	123	10	/	9.8 µW	[81]
	n‐type: a‐IGZO/p‐type: CNT	109	20	/	20 µW	[82]
	n‐type: a‐IGZO/p‐type: CNT	20.9	5	/	1.8 µW	[83]
	n‐type: a‐IZO/p‐type: SWCNT	45	2	2 nW	0.4 µW	[84]
	n‐type: a‐ZSTO/p‐type: SWCNT	41	5	0.78 nW	32 nW	[85]
	n‐type: ZTO/p‐type: SWCNT	17	5	0.25 µW	/	[86]
	n‐type: IGZO/p‐type: SWCNT	1.8	10	/	1.72 µW	[87]

In summary, advances in oxide TFT technology have driven the development of high‐performance, low‐power logic circuits, including NMOS and CMOS inverters, as well as basic logic gates such as NAND and NOR [88, 89, 90, 91]. Integrating these logic circuits offers tremendous potential for low‐cost, flexible, and scalable electronics for a wide range of applications, from wearables to large‐area displays. The high performance of these circuits, especially the high voltage gain, paves the way for their use in energy‐efficient, high‐speed electronic systems.

### 4.2 Active‐Matrix Arrays

Active‐matrix arrays are a key technology in the development of advanced electronics that provide efficient, scalable solutions for display, sensor, and image processing applications [92, 93, 94, 95]. In this field, oxide TFTs emerge as the ideal backplane technology for achieving high‐efficiency, high‐density arrays due to their high mobility, extremely low off‐state current, and compatibility with low‐temperature processes. Their low off‐state current directly reduces the array's static power consumption, while high mobility supports rapid signal read/write operations, contributing to optimized dynamic power consumption at the system level.

**FIGURE 6:**
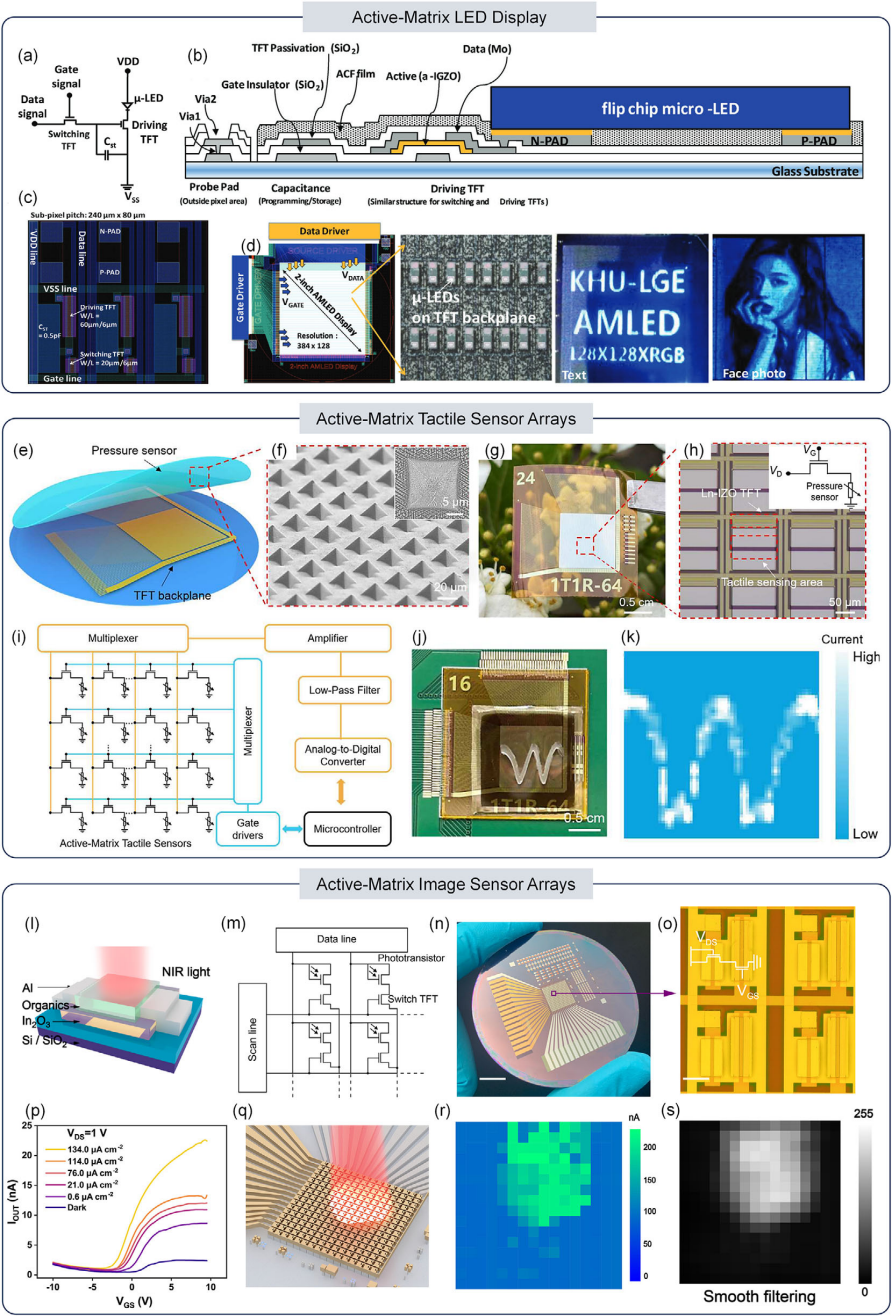
(a) Schematic diagram of the 2T1C pixel circuit in an AMLED display. (b) Cross‐sectional schematic diagram of the TFT‐LED pixel structure. (c) Optical image of the fabricated AMLED array. (d) Optical image of the 2‐inch AMLED display after flip‐chip bonding. Reproduced with permission [96]. Copyright 2018, Wiley‐VCH. (e) Design of the AM‐TSA, which is monolithically integrated with a Ln‐IZO TFT backplane and a pressure‐sensitive film. (f) Tilted SEM image of the 3D CNTs/PDMS film. (g) Photograph of the flexible Ln‐IZO TFT array fabricated on a PI substrate. (h) Enlarged optical microscope image of the Ln‐IZO TFT array, showing an Ln‐IZO transistor and a pressure sensor connected in series. (i) Testing circuit diagram of the tactile sensor array. (j) Photograph of a PDMS mold in the shape of the letter ‘W’ placed on top of the sensor array. (k) Corresponding pressure distribution mapping resulting from the applied pressure. Reproduced with permission [97]. Copyright 2024, IEEE. (l) Schematic illustration of the hybrid phototransistor structure, used for light detection in the array. (m) Equivalent circuit diagram of the hybrid phototransistor array. (n) Digital photograph of the phototransistor array. Scale bar: 1 cm. (o) Optical image showing four pixels of the array. Scale bar: 100 µm. (p) Transfer curves of the switch TFT connected with a hybrid phototransistor under different NIR light intensities. (q) Schematic illustration of the phototransistor array used to image the light distribution from an NIR light beam. (r) Normalized photocurrent mapping of the phototransistor array under NIR light illumination after subtraction of the background noise. (s) Simulated image obtained after spatial smoothing filtering, where the phototransistor array functions as a filter to process the data. Reproduced with permission [98]. Copyright 2022, American Chemical Society.

To meet the demand for low‐power displays with scalable backplanes, Um et al. used flip‐chip bonding to create µLED displays with oxide TFT backplanes, demonstrating a 2‐inch prototype fusion display [96]. The active‐matrix LED (AMLED) display was designed using a conventional 2T1C pixel circuit, which consists of two a‐IGZO TFTs, an LED, and a storage capacitor, as shown in Figure 6a–c. Figure 6d provides an optical image of the 2‐inch AMLED display, showcasing the optical output of text and image, demonstrating its potential for applications not only in large TVs and signages but also in miniature displays for AR/VR and head‐mounted devices.

Beyond displays, tactile interfaces require high‐density, low‐leakage active‐matrix readout to enable power‐efficient large‐area sensing. Tang et al. demonstrated a flexible active‐matrix tactile sensor array (AM‐TSA) with a record‐high pixel density of 4096 pixels/cm^2^ and in‐array sensitivity (51 kPa^−1^) achieved by monolithically integrating an Ln‐IZO TFT backplane with a CNTs/PDMS pressure‐sensitive film [97]. As shown in Figure 6e–f, the AM‐TSA consists of a 64 × 64 Ln‐IZO TFT array and a pressure‐sensitive thin film with 3D CNTs coated PDMS micro‐pyramid and nanopillar structures, which ensures uniform pressure sensing. This highly sensitive sensor film facilitates the integration of high‐density Ln‐IZO TFT arrays (4096 pixels/cm^2^) to create flexible AM‐TSAs, which are essential for haptic robotics applications. Figure 6g shows the fabrication of Ln‐IZO TFT arrays with high mobility and stability on 10 µm thick polyimide (PI) films, while Figure 6h shows an optical image of the one‐transistor‐one‐resistor (1T1R) structure in the pixel array, where the tactile sensor is connected in series. For high‐resolution pressure distribution display, the signal from each pixel of the flexible AM‐TSA is read by row and column scanning. Figure 6i illustrates the readout circuit diagram of the signal acquisition system, with electrodes connected to an external printed circuit board. In Figure 6j, a 3D‐printed mold shaped like the letter “W” is placed on top of the sensor array, with 1 kPa pressure applied. The current distribution image in Figure 6k clearly shows the pressure distribution, demonstrating excellent signal‐to‐noise ratio.

The active‐matrix readout method is also applicable to large‐area flat‐panel imaging. Li et al. developed a dynamic near‐infrared (NIR) sensor array based on solution‐processed In_2_O_3_/BTPV‐4F:PTB7‐Th hybrid phototransistors, which exhibit a strong photogating effect and in‐sensor image preprocessing capabilities [98]. Figure 6l shows the bulk heterojunction hybrid phototransistor structure with bottom‐gate, top‐contact structure. To demonstrate the feasibility of these hybrid phototransistors for large‐area NIR sensing, the authors constructed a 16 × 16 phototransistor array with an area of 1 × 1 cm^2^, where each pixel consists of a hybrid phototransistor and an In_2_O_3_ switching transistor, as shown in Figure 6m. To ensure proper operation in the saturation region, the gate and drain of the phototransistor are connected together. The digital photograph and optical image showing the array and a top view of four pixels are presented in Figure 6n,o, respectively. The switching TFT in each pixel maintains the I_OFF_ value around 1 nA, as shown in Figure 6p, which effectively suppressed static power consumption in the dark. Under the NIR illumination, the on‐state current is dominated by the phototransistor, and as the optical power increases, the resistance of the phototransistor decreases, leading to an increase of the on‐state current. Figure 6q‐s show the NIR beam schematic and current mapping, demonstrating the feasibility of using the hybrid phototransistor in real‐time dynamic NIR image sensors. Given that the fabrication process is compatible with standard semiconductor techniques, future advancements will likely achieve even higher pixel densities in phototransistor arrays.

In conclusion, the integration of active‐matrix arrays for LED displays, tactile sensors, and image sensors represents a significant leap forward in electronic device design [99, 100]. These arrays, driven by the innovation of oxide TFTs, provide enhanced performance in terms of low power consumption, resolution, sensitivity.

### 4.3 Flexible Electronics

Flexible electronics (e.g., flexible tactile sensors, phototransistors, biosensors and circuits, etc.) are of interest due to their potential for a wide range of applications such as wearable devices, health monitoring, human‐machine interfaces and environmental sensing [101]. These application scenarios often require devices to operate for extended periods under battery power or energy‐constrained conditions. Oxide TFTs are well suited for flexible electronics due to their high mobility, low off‐state current, and low‐temperature processing characteristics [102, 103, 104].

**FIGURE 7:**
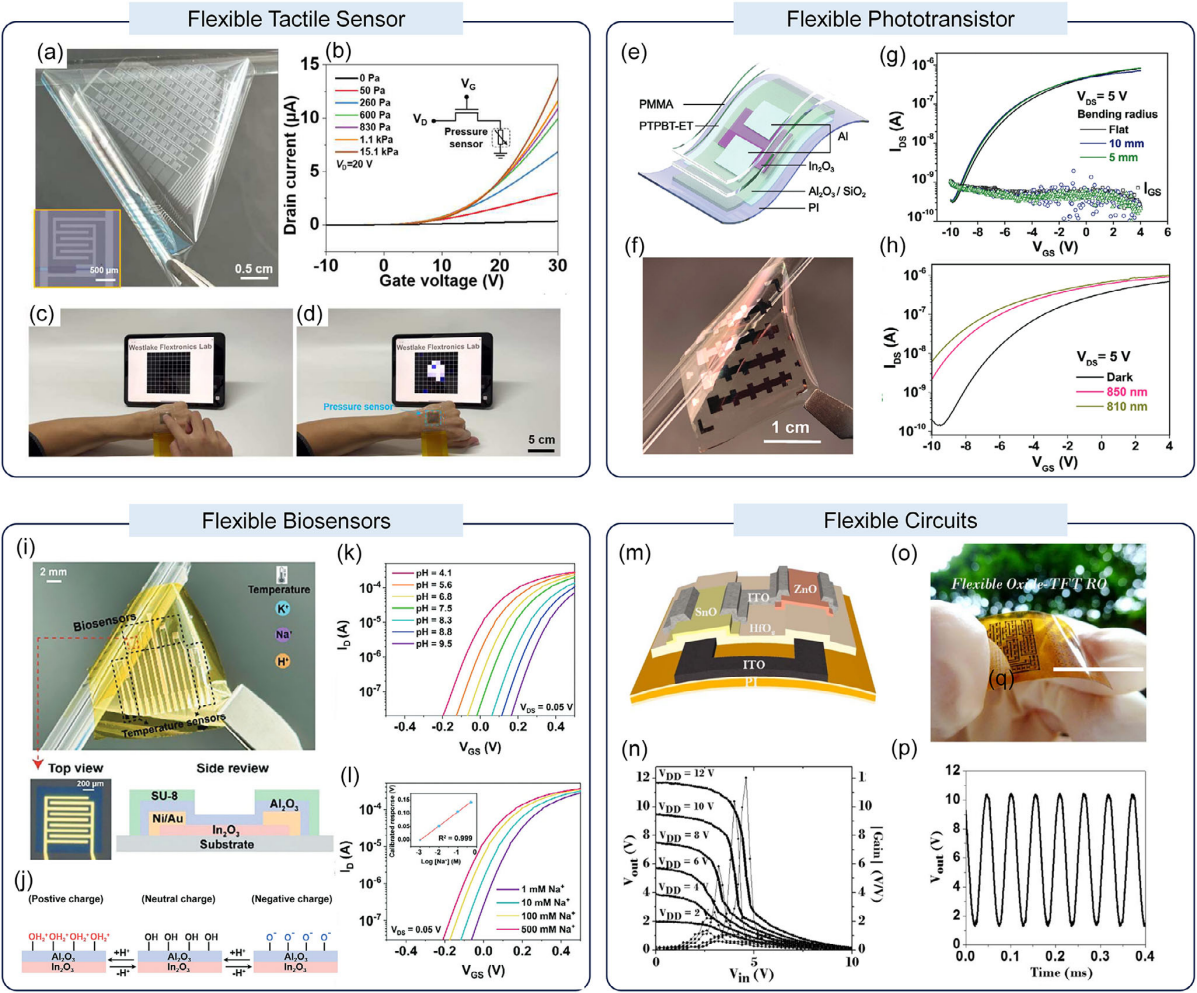
(a) Photograph of the flexible and transparent AM‐TSA. (b) Transfer curves of a single 1T1R sensor pixel. (c, d) Photographs demonstrating real‐time visualization of pressure distribution. Reproduced with permission [105]. Copyright 2022, IEEE. (e) Schematic diagram of the flexible In_2_O_3_/PTPBT‐ET hybrid phototransistor structure. (f) Photograph showing the flexible hybrid phototransistor device. Scale bar: 1 cm. (g) Transfer curves of the flexible device in flat status and under bending. (h) Transfer curves of the flexible device under various NIR light intensities after 1000 bending cycles. Reproduced with permission [106]. Copyright 2021, Wiley‐VCH. (i) Photograph of the flexible Al_2_O_3_/In_2_O_3_‐based bio‐FET array. (j) Schematic illustration of the pH response mechanism. (k) Typical transfer characteristics of the flexible Al_2_O_3_/In_2_O_3_ bio‐FET under different pH levels. (l) Transfer curves of the flexible Na^+^ ion‐selective membrane‐based bio‐FET under varying Na^+^ concentrations. Reproduced with permission [107]. Copyright 2024, Wiley‐VCH. (m) Schematic view of a flexible CMOS inverter based on an n‐channel ZnO TFT and a p‐channel SnO TFT. (n) Static voltage transfer characteristics and voltage gain curves of the flexible CMOS inverter at different V_dd_. (o) Flexible oxide‐TFT‐based CMOS ring oscillators fabricated on a polyimide foil substrate. (p) Output waveform of a five‐stage flexible CMOS ring oscillator. Reproduced with permission [109]. Copyright 2016, IEEE.

Monolithically integrating oxide backplanes with pressure‐sensitive materials enables high‐performance wearable interfaces. Tang et al. demonstrated a wearable, transparent AM‐TSA by monolithically integrating an In_2_O_3_ TFT backplane with a highly pressure‐sensitive micro‐pyramidal film on colorless PI (Figure 7a) [105]. The inset image provides an optical view of a single integrated 1T1R sensor pixel, which consists of interleaved electrodes from an In_2_O_3_ TFT and a resistive tactile sensor. The inherently low off‐state current of the oxide TFT backplane ensures extremely low static power consumption and avoids signal crosstalk in the large‐scale sensor array. Figure 7b illustrates the equivalent circuit diagram and transfer curves at various external pressures (0–15.1 kPa). This AM‐TSA allows for real‐time mapping of tactile pressure distribution as shown in Figure 7c,d, providing a feasible solution for low‐power electronic skin.

Extending functionality to optical perception, Li et al. fabricated flexible In_2_O_3_/PTPBT‐ET phototransistors on PI substrates, as shown in Figure 7e [106]. Figure 7f shows a photograph of the flexible phototransistor after it was peeled off from the carrier glass. These flexible phototransistor devices exhibit stable electrical performance at various bending radii (as small as 5 mm), as seen in Figure 7g, where performance degradation is minimal even after 1000 bending/releasing cycles. Additionally, Figure 7h shows the optical response of the flexible phototransistor to 810 and 850 nm NIR light after 1000 bending cycles, demonstrating significant NIR response and robust mechanical flexibility. These characteristics pave the way for the use of In_2_O_3_/PTPBT‐ET hybrid phototransistors in NIR sensing for portable and wearable interfaces.

Specifically for bio‐signal sensing, implementing liquid gating allows for a substantial reduction in operating voltage, thereby further suppressing power consumption. Ren et al. developed a fully integrated wearable biosensing system for acquiring physiological signals [107]. This system integrates a multiplexed FET sensor array on a flexible substrate (Figure 7i) for detecting ions such as H^+^, Na^+^, K^+^, and temperature. Its sensing mechanism relies on protonation or deprotonation reactions occurring on the surface of the Al_2_O_3_/In_2_O_3_ films, which is illustrated in Figure 7j. Figure 7k presents the typical transfer curves of a flexible biosensor in pH 4 to 10 buffer solutions. Figure 7l shows the transfer characteristics of the biosensor with a Na^+^ ion‐selective membrane across various NaCl concentrations. These characteristics make it particularly suitable for long‐term, dynamic monitoring of health metrics.

In the field of flexible logic circuits, the core to achieving low power consumption lies in adopting CMOS architectures, which fundamentally reduce static power consumption [108]. Li et al. demonstrated flexible CMOS inverters utilizing p‐type SnO and n‐type ZnO channels, as illustrated in Figure 7m,n [109]. Moving beyond individual logic gates to integrated circuits, a flexible five‐stage ring oscillator was successfully fabricated (Figure 7o). The oscillator generates stable output waveforms (Figure 7p), confirming that the low‐power advantages and integration capability of oxide‐based CMOS technology are well‐preserved in flexible form factors.

In summary, flexible electronics based on oxide TFTs have shown remarkable potential across various applications such as wearable sensors, phototransistors, biosensors, and logic circuits. The integration of high‐performance oxide materials into flexible TFTs has enabled the development of highly sensitive, durable, and efficient devices, which are critical for real‐time sensing, health monitoring, and human‐computer interaction.

### 4.4 Monolithic 3D Integration

Monolithic three‐dimensional (M3D) integration is a promising approach to overcoming the limitations of traditional scaling of semiconductor devices, achieving high‐density integration, improved performance, and reduced power consumption [110, 111, 112, 113]. By stacking multiple layers of functional devices, 3D integration enables more compact and efficient circuits while minimizing interconnect distances, resulting in significantly lower power loss and higher data throughput [114, 115, 116].

**FIGURE 8:**
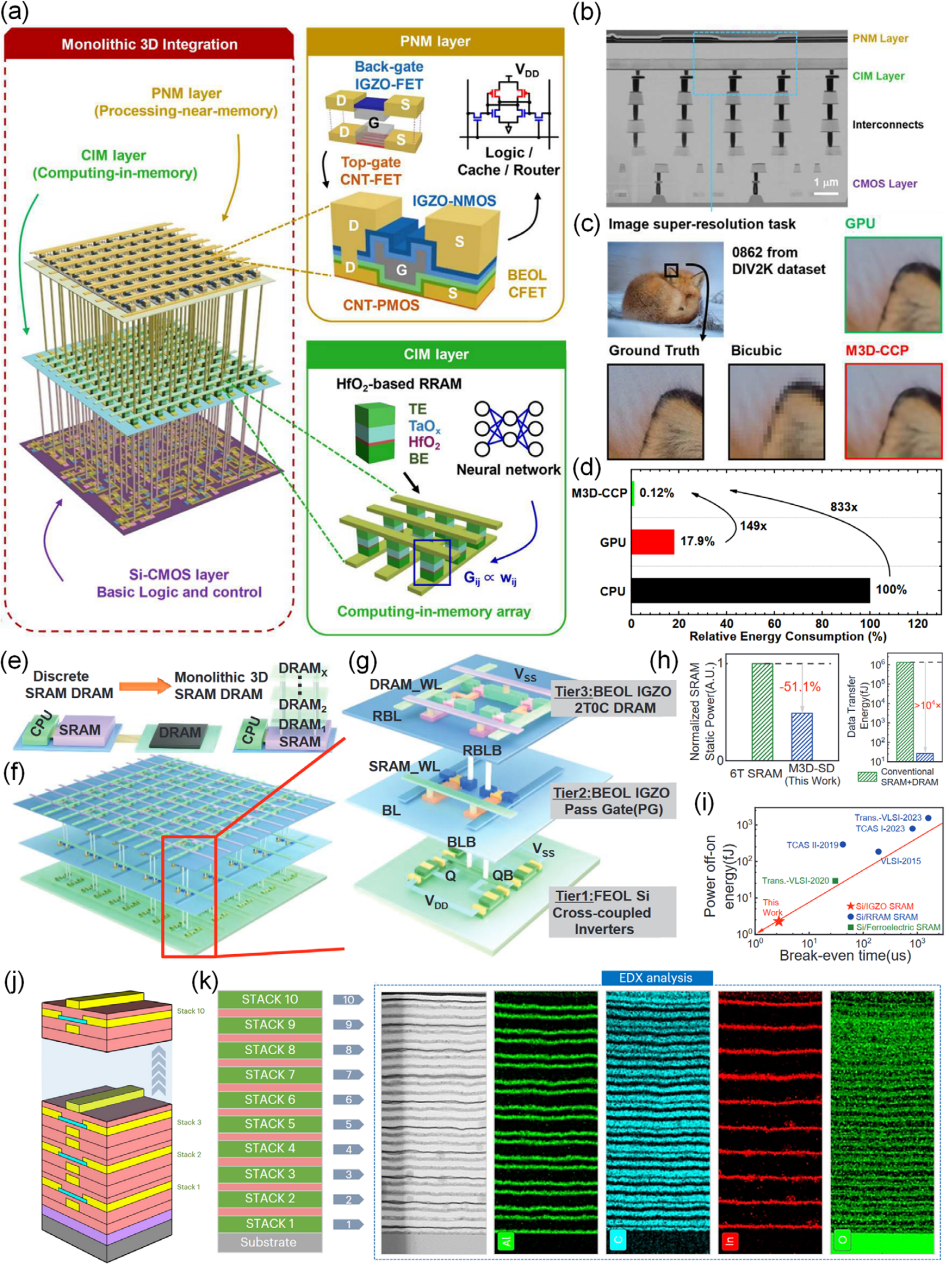
(a) Architecture of the M3D‐CCP chip with three layers: Si CMOS for control logic, CIM layer with HfO_2_‐based RRAM for MVM, and PNM layer using complementary‐FET for memory and data processing. (b) Cross‐sectional TEM images of the M3D‐CCP chip demonstrated in this work. (c) Implementation of the neural network for image super‐resolution using the M3D‐CCP chip architecture, as demonstrated in this work. (d) Energy consumption benchmark for the M3D‐CCP, GPU, and CPU, highlighting the energy efficiency of the M3D‐CCP chip. Reproduced with permission [117]. Copyright 2022, IEEE. (e) Motivation of the M3D‐CCP design for high‐performance computing and memory integration. (f,g) Architecture of the M3D‐SRAM‐DRAM chip, achieved by 3D integration of Tiers 1–3, showing the integration of memory layers. (h) Left: static power comparison of the 6T SRAM and this proposed M3D‐CCP. Right: Data transfer energy comparison of the conventional SRAM+DRAM and this proposed M3D‐CCP. (i) Benchmark of power‐off/on energy for SRAMs, demonstrating the superior performance of the M3D‐SD cell. Reproduced with permission [119]. Copyright 2024, IEEE. (j) 3D schematic representation of 10‐stack DG In_2_O_3_ transistors on a Si/SiO_2_ substrate. (k) Schematic depiction of the 10‐stack structure. Cross‐sectional transmission electron microscopy images (left) and energy‐dispersive X‐ray spectroscopy analysis (right) of the completed 10‐S device, showing the distribution of Al, C, In, and O atoms across the device layers. Reproduced with permission [61]. Copyright 2024, Springer Nature.

In computing‐in‐memory (CIM) architectures, 3D integration enables the tight coupling of memory and processing units, fundamentally mitigating the energy overhead of the memory wall. An et al. proposed a 3D integrated hybrid CIM architecture, named M3D‐CCP, as shown in Figure 8a,b [117]. The architecture consists of three layers: the first layer is a Si‐CMOS circuit, used for basic control and logic; the second layer is a CIM layer for matrix‐vector multiplication (MVM) in neural networks; and the third layer is a processing‐near‐memory (PNM) layer based on CNT‐PMOS and IGZO‐NMOS, which is used for caching and processing data in neural networks. In an image super‐resolution task (Figure 8c), this chip achieved accuracy comparable to a GPU while consuming 149 times and 833 times less energy than a GPU and a CPU, respectively (Figure 8d), highlighting the profound advantage of 3D integration in curbing data‐movement energy [118].

Similarly, 3D integration provides an ideal platform for constructing high‐efficiency, high‐density memory hierarchies. To address the density and power limitations of conventional SRAM, Liu et al. proposed an M3D integrated circuit platform for stacked SRAM and DRAM (SD), as shown in Figure 8e [119]. The M3D‐SD architecture consists of ultra‐high‐density IGZO/Si SRAM and IGZO‐based 2T0C DRAM in a three‐tier structure (Figure 8f,g). Crucially, this hybrid design leverages the unique properties of IGZO TFTs to reduce power consumption. In the SRAM tier (Tier‐2), the replacement of conventional silicon pass‐gates with wide‐bandgap IGZO TFTs effectively suppresses subthreshold conduction and gate‐induced drain leakage, directly reducing the static standby power by 51.1% compared to conventional 6T SRAM (Figure 8h, left). Furthermore, the exceptional off‐state performance of the IGZO 2T0C DRAM tier (Tier‐3) enables ultra‐long data retention (> 2100 s), thereby eliminating the power consumption typically required for periodic refresh in conventional DRAM. Simultaneously, the vertical stacking geometry eliminates long off‐chip data buses and minimizes parasitic interconnect capacitance, thereby slashing the SRAM‐to‐DRAM data transfer energy by over 10^4^ times compared to discrete systems (Figure 8h, right) and attaining a recordlow datatransfer energy of 2.26 fJ (Figure 8i). These results conclusively demonstrate the pivotal role of oxide TFT technology in realizing low‐power M3D integrated memory systems.

Further, Yuvaraja et al. tackled scalable process development for integrating 3D TFT devices by monolithically stacking 10 n‐type In_2_O_3_ TFTs with different architectures (BG, TG and DG), as shown in Figure 8j [61]. This process is realized by sequentially processing several layers of functional devices at near‐room temperature. Figure 8k presents cross‐sectional transmission electron microscopy and energy‐dispersive X‐ray spectroscopy images, confirming conformal deposition of the layers and minimal interlayer diffusion, essential for good 3D vertical integration and optimal device performance.

In summary, monolithic 3D integration, particularly using oxide TFTs, is a powerful technique for improving device performance, increasing data bandwidth, and enhancing the density of integrated circuits [120, 121]. The M3D‐CCP architecture, 3D memory systems, and 3D integration of oxide TFTs showcase the potential of this technology for advancing high‐performance electronics. The ability to stack multiple functional layers not only increases device density but also enables new functionalities such as efficient memory computing, low latency, and ultra‐low power consumption.

### 4.5 Neuromorphic Electronics

Neuromorphic electronics aims to emulate biological nervous system by mimicking neuronal spiking and synaptic plasticity, enabling brain‐inspired computing with parallel processing, adaptive learning, and low power consumption [122, 123, 124]. Oxide TFTs are ideal candidates for neuromorphic devices due to their high carrier mobility, low power consumption, and large‐area uniformity. These features allow oxide TFTs to drive innovations in neuromorphic systems, particularly in simulating synaptic weight changes and achieving neuronal pulse timing‐dependent plasticity, which mimics biological learning, memory, and forgetting [125, 126, 127, 128, 129].

**FIGURE 9:**
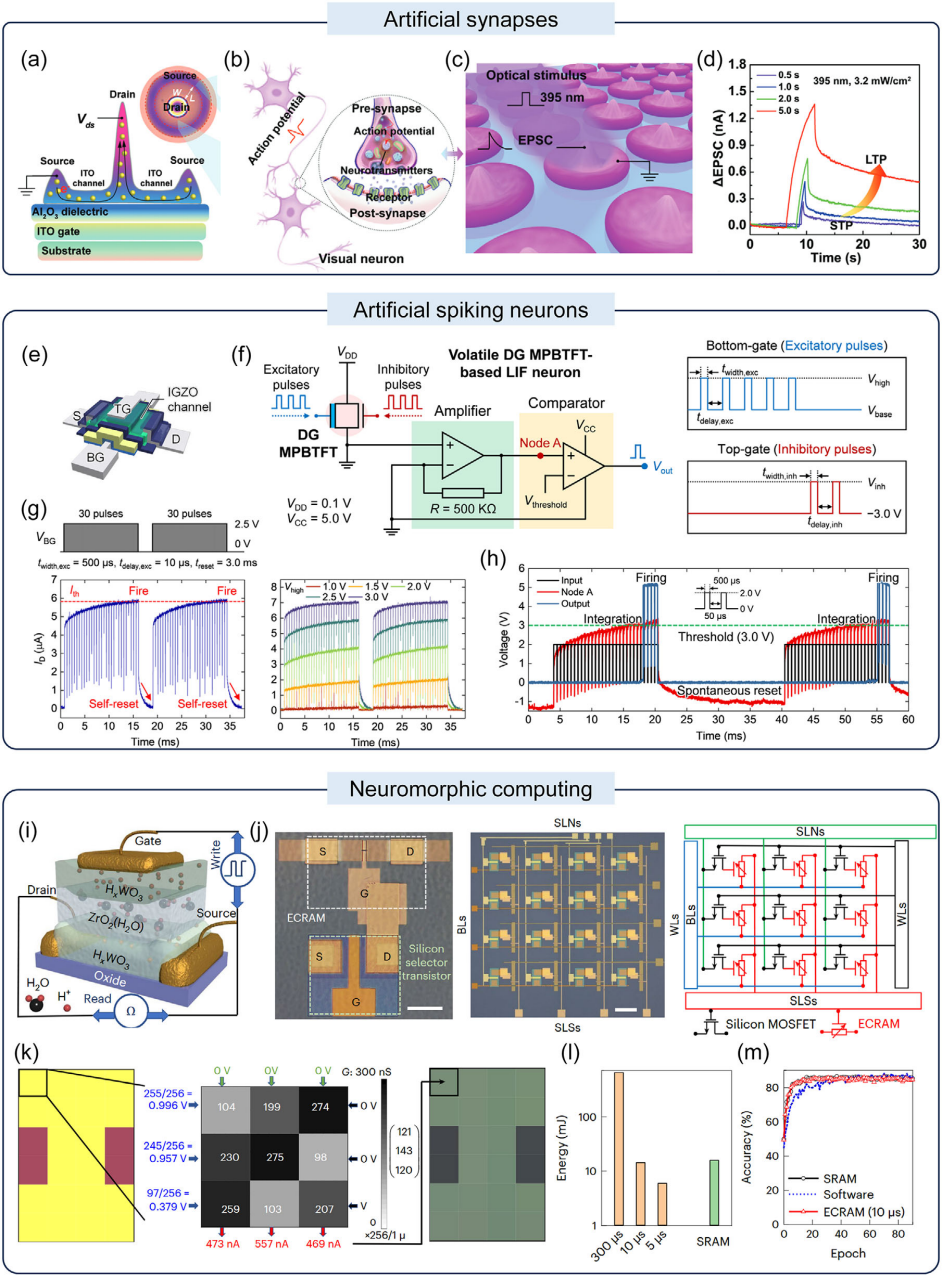
(a) Schematic cross‐section of the printed coffee‐ring transistor. The inset shows a top view of the coffee‐ring transistor structure. (b, c) Schematic diagrams for simulating a biological synapse (b) using a synaptic coffee‐ring transistor (c) under the coordinated modulation of optical and electrical inputs. (d) Transition from short‐term plasticity to long‐term plasticity by increasing the duration of optical pulses. Reproduced with permission [130]. Copyright 2022, Wiley‐VCH. (e) Schematic of a volatile DG MPBTFT enabling spiking‐neuron operation. (f) DG MPBTFT‐based LIF neuron circuit and the corresponding excitatory (bottom‐gate) and inhibitory (top‐gate) pulse schemes. (g) Measured current responses showing neuron firing/self‐reset behavior and tunability under different excitation conditions. (h) Neuronal behavior of DG MPBTFT‐based LIF neuron. Reproduced with permission [131]. Copyright 2024, Springer Nature. (i) Device schematic of a monolithic all‐solid‐state protonic electrochemical random‐access memory (ECRAM). (j) Optical micrograph of an ECRAM cell (left) and an integrated pseudo‐crossbar array with silicon selector transistors (middle), together with the circuit schematic (right). (k) Example of in‐memory operation using the ECRAM array. (l) energy consumption for the ECRAM‐CMOS hybrid in‐memory computing accelerator to learn the classification of the MNIST dataset using different write‐pulse widths (orange), as benchmarked against the SRAM‐based digital accelerator or software (green). (m) Simulated accuracy of ECRAM‐based (red), SRAM‐based (black) accelerators and software (blue dotted line) to learn the classification. Reproduced with permission [132]. Copyright 2024, Springer Nature.

Artificial synapses are one of the basic neuromorphic devices. Liang et al. reported a printed all‐oxide optoelectronic synaptic transistor array with high pixel density and uniformity, based on a modified coffee‐ring structure (Figure 9a,b) [130]. In this device, UV light pulses serve as the presynaptic input, and the induced channel current changes mimic biological excitatory postsynaptic currents (Figure 9c,d). This light‐controlled synaptic plasticity originates from the ionization and recombination of oxygen vacancies in the ITO film, which behaves similarly to biological memory loss. This inherent mechanism exhibits extremely low static power consumption, providing a device‐level foundation for constructing large‐area, low‐energy artificial visual perception networks.

Artificial spiking neurons are another type of neuromorphic device. Kim et al. demonstrated a volatile DG ferroelectric‐to‐mixed phase boundary TFT (MPBTFT), which composed of hafnium zirconium oxide (HZO) and IGZO channel to realizes a leaky integrate‐and‐fire (LIF) neuron, as shown in Figure 9e [131]. By harnessing the volatile polarization and partial switching characteristics of the HZO ferroelectric layer, the device intrinsically emulates the neuronal dynamics of integration, firing, and self‐reset via its inherent leakage (Figure 9f–h). Crucially, a single DG MPBTFT can process excitatory and inhibitory pulses separately through its bottom and top gates, eliminating the need for external capacitors and reset circuits. This greatly simplifies the architecture and removes associated power overhead at the device level.

As for neuromorphic computing systems, in‐memory computing architectures offer a fundamental pathway to ultra‐low power consumption by eliminating data movement. Cui et al. proposed the oxide‐semiconductor‐based electrochemical random‐access memory (ECRAM) [132]. As shown in Figure 9i, this all‐inorganic protonic ECRAM utilizes an ion‐intercalation mechanism to achieve analog, non‐volatile weight storage, enabling the direct execution of multiply‐accumulate operations within the memory cells themselves. To ensure compatibility with standard silicon circuits and achieve high‐density integration, this ECRAM was integrated with a silicon selector transistor (Figure 9j, left). A pseudo‐crossbar array constructed on this basis can efficiently perform parallel vector‐matrix multiplication (Figure 9j, middle and right). As demonstrated in a color transformation example (Figure 9k), analog input voltages are processed directly through the array, with results output in the form of current, thereby entirely avoiding the energy overhead associated with reading and moving digital weights. Meanwhile, the overall energy consumption is up to two times lower than SRAM‐based accelerators (Figure 9l), while maintaining high accuracy in tasks such as the image classification training shown in Figure 9m.

In summary, neuromorphic electronics based on oxide TFTs, are enabling the development of advanced devices that mimic the functionality of biological neural systems. These devices, such as artificial synapses and spiking neurons, demonstrate remarkable potential in simulating learning, memory, and biological signaling processes [129, 133, 134]. Moreover, the integration of oxide TFTs in neuromorphic systems allows for low‐power and high‐accuracy capabilities in areas of intelligent perception. As neuromorphic devices continue to evolve, they hold promise for further pushing the boundaries of brain‐like computing and adaptive learning [135, 136, 137].

## 5 Conclusion and Outlook

**FIGURE 10:**
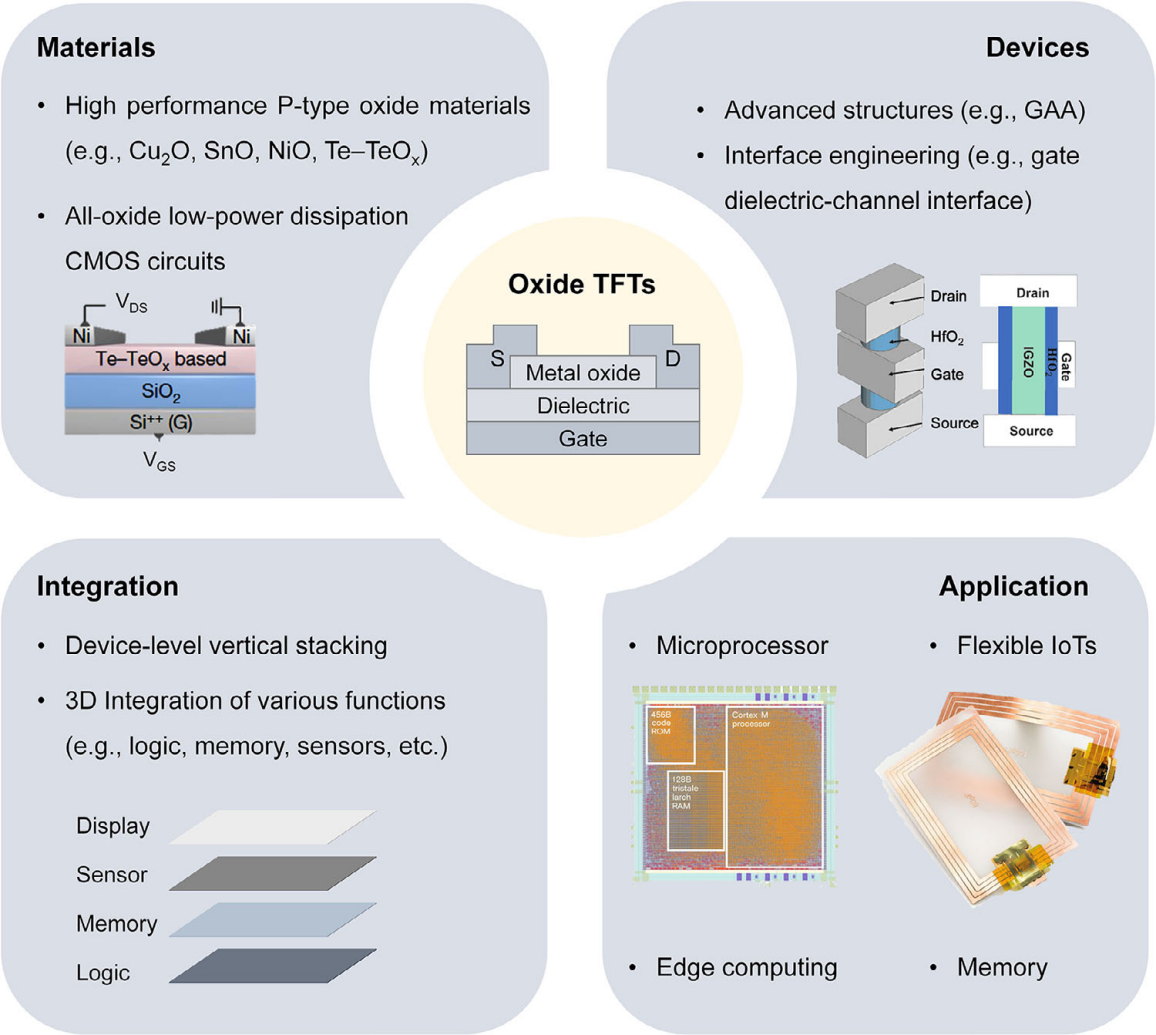
Future directions for oxide TFTs. This diagram highlights the potential of oxide TFTs in future electronics, focusing on high‐performance p‐type materials, advanced device structures and interface engineering, 3D integration, and applications in microprocessor, flexible IoTs, memory, and edge computing. Reproduced with permission [72]. Copyright 2024, Springer Nature. Reproduced with permission [144]. Copyright 2025, Springer Nature. Reproduced with permission [155]. Copyright 2021, Springer Nature. Reproduced with permission [17]. Copyright 2018, Springer Nature.

This review provides an overview of advances in oxide TFTs for low‐power electronics, focusing on their material properties, methods to reduce power consumption, and their applications in various field. First, the inherent advantages of oxide materials, such as high electron mobility, low off‐state current, and excellent large‐area uniformity, are emphasized. These properties make oxide TFTs ideal candidates for low‐power electronic devices, thus enabling efficient operation in energy‐constrained applications. Next, strategies to reduce the power consumption of oxide TFTs are discussed in depth, focusing on interface engineering and structural engineering. Interface engineering, including optimization of the semiconductor‐electrode interface and optimization of electrode characteristics, has been shown to significantly reduce leakage current and improve device stability. Structural engineering, such as UL and DG structures, further optimizes electrical performance by reducing parasitic capacitance and improving the switching characteristics of the transistor. These engineering approaches synergistically reduce static and dynamic power consumption, making oxide TFTs more suitable for low‐power applications. Finally, a wide range of applications for oxide TFTs in low‐power electronics, including logic circuits, active‐matrix arrays, flexible electronics, M3D integration, and neuromorphic computing, are also discussed. Despite the significant progress made in oxide TFT technology, there are still several challenges and opportunities for further development in this field (Figure 10).

1. Material development: One of the main limitations of oxide TFTs is the lack of reliable p‐type oxide semiconductors. [138, 139, 140, 141] The absence of p‐type devices means current oxide circuits mainly rely on unipolar or pseudo‐complementary logic (such as depletion‐mode load inverters), which introduces a direct current path in at least one logic state and thus leads to appreciable static power consumption. In contrast, p‐type oxide TFTs would enable the realization of fully complementary oxide CMOS, bringing static power consumption closer to leakage‐limited levels. Consequently, developing high‐performance p‐type oxide semiconductors is a core prerequisite for achieving low power consumption circuits [142].
2. Device optimization: At the device level, reducing power consumption requires coordinated advances in device architecture and interface engineering. On one hand, advanced structures such as gate‐all‐around (GAA) devices can enhance gate control capabilities, effectively suppressing leakage current to reduce static power consumption; simultaneously, near‐ideal SS can be achieved, enabling significant reduction of V_dd_ and directly cutting dynamic power consumption [143, 144]. On the other hand, by optimizing interfaces such as the gate dielectric‐channel interface and the source/drain contact‐semiconductor interface, it not only significantly suppresses off‐state leakage current to lower static power consumption, but also enhances gate control capability and carrier transport efficiency, thus boosting current drive capability at the same voltage and optimize dynamic power consumption.
3. Integration optimization: At the system integration level, vertical stacking and multi‐layer integration technologies are important pathways for reducing power consumption [145, 146]. This technology effectively shortens interconnect lengths and reduces parasitic resistance and capacitance, thereby directly lowering dynamic power consumption caused by interconnections. Furthermore, the integration of multiple functions such as memory, logic, and sensors within stacked 3D architectures enables near‐sensor computing, significantly reducing data transport power consumption between modules [147, 148, 149, 150, 151]. Although this technology still faces challenges in process compatibility, thermal management, and reliability, 3D integration provides a critical structural foundation for achieving low‐power electronic systems.
4. Application expansion: Oxide TFTs hold significant promise in low‐power electronics. Beyond their established applications in logic circuits, flexible electronics and brain‐inspired computing, numerous emerging application scenarios remain to be explored. Based on the inherent low off‐state current, favorable subthreshold characteristics, and compatibility with flexible substrates, this technology demonstrates significant potential across multiple areas including microprocessors [152, 153, 154, 155], memory [156, 157, 158], flexible IoTs, and edge computing [159]. The low‐power characteristics of oxide TFTs provide critical support for these emerging fields. Their ultra‐low static power consumption enables long‐term standby operation, while their outstanding dynamic energy efficiency reduces data processing overhead in edge computing.

## Conflicts of Interest

The authors declare no conflicts of interest.

## Data Availability

The data that support the findings of this study are available from the corresponding author upon reasonable request.
